# Changing Trends of Cirrhotic and Noncirrhotic Hepatocellular Carcinoma in the Era of Directly-Acting Antiviral Agents

**DOI:** 10.14309/ctg.0000000000000420

**Published:** 2021-11-03

**Authors:** Karan Mathur, Areej Mazhar, Milin Patel, Lara Dakhoul, Heather Burney, Hao Liu, Lauren Nephew, Naga Chalasani, Andrew deLemos, Samer Gawrieh

**Affiliations:** 1 Division of Gastroenterology & Hepatology, Department of Medicine, Indiana University School of Medicine, Indianapolis, Indiana, USA;; 2 Department of Medicine, Atrium Health, Charlotte, North Carolina, USA;; 3 Department of Gastroenterology & Hepatology, Roudebush VA Medical Center, Indianapolis, Indiana, USA;; 4 Department of Biostatistics and Health Data Science, Indiana University School of Medicine, Indianapolis, Indiana, USA.

## Abstract

The impact of direct-acting antivirals (DAAs) for hepatitis C virus (HCV) on burden of cirrhotic and noncirrhotic hepatocellular carcinoma (HCC) has not been examined. We assessed recent trends in liver disease etiologies of HCC and proportion of noncirrhotic HCC since DAAs introduction.

**METHODS:**

Clinical characteristics including presence or absence of underlying cirrhosis were collected from 2,623 patients diagnosed with HCC between 2009 and 2019 at 2 large US centers. Logistic regression was performed to investigate the annual trends of HCC due to different liver diseases and proportions of noncirrhotic cases.

**RESULTS:**

In the DAA era (2014–2019), annual decline in HCV-HCC (odds ratio [OR] = 0.93, 95% confidence interval [CI] 0.88–0.99, *P =* 0.019), without change in trends of other liver diseases–related HCC, was observed. Annual increase in noncirrhotic HCC (OR 1.13, 95% CI 1.03–1.23, *P =* 0.009) and decline in cirrhotic HCC (OR 0.89, 95% CI 0.81–0.97, *P =* 0.009) along with similar trends for HCV-HCC—increase in noncirrhotic cases (OR 1.35, 95% CI 1.08–1.69, *P =* 0.009) and decrease in cirrhotic cases (OR 0.92, 95% CI 0.86–0.98, *P =* 0.012)—were observed during the DAA era. Compared with the pre-DAA era, HCC resection rate increased (10.7% vs 14.0%, *P =* 0.013) whereas liver transplantation rate decreased (15.1% vs 12.0%, *P =* 0.023) in the DAA era.

**DISCUSSION:**

Since introduction of DAAs, proportions of cirrhotic HCC have decreased, whereas proportions of noncirrhotic HCC have increased. These new trends were associated with change in utilization of liver resection and transplantation for HCC. The impact of changing patterns of DAA use on these trends will require further study.

## INTRODUCTION

Hepatocellular carcinoma (HCC) is the fourth most common cause of cancer-related mortality worldwide (1). In the United States, incidence rates of HCC increased by 4.5% per year from 2000 to 2009 and then slowed down to rise 0.7% annually until the year 2012 (2). In North America, the age-standardized incidence rates of HCC have increased more than 100 percent between 1990 and 2015 (3). HCC-associated deaths are forecasted to be significantly worse in 2040 compared with 2016 (4). Chronic hepatitis C virus (HCV), chronic hepatitis B virus (HBV), and alcoholic liver disease are the most common underlying etiologies for HCC globally (1,5).

HCC secondary to HCV (HCV-HCC) occurs mostly in the background of cirrhosis (6). The introduction of direct-acting antivirals (DAAs) treatment in 2014 revolutionized the management of HCV. More than 90% of patients treated with DAA achieve sustained virological response (SVR) regardless of fibrosis stage (7). Even patients with decompensated cirrhosis have excellent SVR rates (8). Despite initial controversy, the impressive SVR rates associated with DAA seem to decrease but not completely eliminate the risk of HCV-HCC (9–12). The 2- to 3-year incidence of HCC is estimated to be 3%–4% after achieving SVR with DAA therapy (10,12). There have been concerns on whether DAA treatment may be a risk for development of *de novo* HCC after achieving SVR (13,14). However, several reports and expert consensus have demonstrated the case to be otherwise (12,15–19).

The burden of nonalcoholic fatty liver disease (NAFLD)-related HCC (NAFLD-HCC) in the United States has increased in recent years (20). In the 2004–2009 surveillance, epidemiology, and end results Medicare database study, NAFLD was the third leading cause of HCC after HCV and alcohol (21), trends that we confirmed in a recent large multicenter study (22). In addition, between 2000 and 2014, we observed an increased proportion of cirrhotic HCC and a decline in noncirrhotic HCC mainly because of significant annual increases in cirrhotic HCC due to HCV and NAFLD (22). It is unclear whether these trends have changed since the introduction of DAAs for HCV in 2014, especially with the continued rise in NAFLD burden in the United States (23). In this study, we aimed to evaluate trends of underlying liver diseases associated with HCC and underlying cirrhotic status at the time of HCC diagnosis since introduction of DAAs. We also examined whether there had been associated changes in utilization of different HCC treatment modalities in the DAA era. We used patient-level data from 2 large US centers 5 years before and 5 years after the introduction of DAA therapy.

## METHODS

### Patient identification and characterization

This was a retrospective study involving 2 academic tertiary care medical centers: Indiana University School of Medicine, Indianapolis, Indiana, and Atrium Health, Charlotte, North Carolina. The study protocol was approved by the Institutional Review Board of both participating sites. We addressed all the important aspects of the Strengthening the Reporting of Observational studies in Epidemiology statement guidelines (24).

Using the institutional cancer registry, patients diagnosed with HCC between January 2009 and June 2019 were identified. A manual chart review of each HCC case was completed using the electronic medical record to verify the HCC diagnosis. Confirmation of HCC diagnosis was performed either by histological or radiological evidence as per the American Association for Study of Liver Disease guidelines (25). Clinical data at the time of diagnosis, including demographics, comorbidities, date of HCC diagnosis, underlying HCC etiology, laboratory values, presence or absence of cirrhosis, method of diagnosis, and surveillance status, were collected. Histology and radiology data were used to determine tumor size, vascular invasion, staging, and Milan status. Histological grading of HCC on pathology was defined as poorly differentiated, moderately differentiated, well-differentiated, or anaplastic. The Tumor, Node, Metastasis classification was used for anatomic staging (26). We also used the Milan criteria to define the tumor burden regarding size and number of lesions (27). Clinical staging at the time of HCC diagnosis was conducted using the Barcelona Clinic Liver Cancer staging criteria (28). All treatment modalities received for HCC during the disease course were also identified. These modalities were classified as resection, liver transplantation, catheter-directed therapy, radiofrequency ablation, microwave ablation, stereotactic body radiation therapy, sorafenib, palliative/hospice care, other (systemic agents), none, and unknown. For HCV patients, data on all treatment including DAA therapy were collected. Cases were classified as DAA therapy recipients if they took any of the following medications alone or in combination: sofosbuvir, simeprevir, daclatasvir, elbasvir, ledipasvir, glecaprevir, grazoprevir, voxilaprevir, ombitasvir, paritaprevir, ritonavir, dasabuvir, and/or velpatasvir. Of note, although sofosbuvir became available for clinical use after its approval by the US Food and Drug Administration in December 2013, some of our patients had access to it before that as part of participation in clinical trials. Information was also obtained on whether patients achieved posttreatment SVR. Patients diagnosed with HCC before the year 2015 were included in other studies performed by this group (22,29,30). The database was managed centrally using the REDCap secure web application.

### Determination of comorbidities and underlying HCC etiology

Chart documentation and/or laboratory confirmation was used to verify other comorbidities, including hypertension, diabetes mellitus, dyslipidemia, coronary artery disease, peripheral vascular disease, HIV, and history of alcohol abuse. Underlying HCC etiologies were classified as HCV, HBV, alcohol, NAFLD, autoimmune hepatitis, primary biliary cholangitis, primary sclerosing cholangitis, hemochromatosis, alpha1 antitrypsin deficiency, rare, and unclear/unknown. The rare disease category comprised amyloidosis, sarcoidosis, cardiac cirrhosis, drug-induced liver disease, and environmental exposure. HCC etiology was classified as unclear/unknown if no clear underlying chronic liver disease was identified or if there were insufficient data to make the diagnosis. The criteria for alcohol abuse were based on consumption of more than 3 drinks per day, documentation of alcoholism/alcohol abuse in the records, history of alcoholic hepatitis, or participation in an alcohol abuse treatment program (31). The diagnosis of NAFLD was made on the basis of electronic medical record documentation of managing physician and/or presence of hepatic steatosis on radiology or histology without evidence of excessive alcohol use or alternative liver disease. For HCC cases with a combination of underlying viral hepatitis along with alcohol abuse, primary etiology of chronic liver disease was assigned as viral hepatitis.

### Determination of cirrhotic and noncirrhotic status

HCC cases were divided into 4 categories based on the criteria developed by Mittal et al. (31) and validated by our group (22): category 1: level 1 evidence (very high probability) of no cirrhosis, confirmed by both histological and radiological evidence; category 2: level 2 evidence (high probability) of no cirrhosis, based on imaging and laboratory criteria in the absence of histology; category 3: confirmed cirrhosis, based on histological, imaging, clinical, or laboratory criteria; and category 4: unclassified, cirrhosis categories could not be delineated because of insufficient data. Based on the laboratory values, the model of end-stage liver disease score, fibrosis-4 score, and the aspartate aminotransferase-to-platelet ratio index were also calculated. For cirrhotic cases, information on any liver-related complications such as ascites, varices, hepatic encephalopathy, and spontaneous bacterial peritonitis since the date of HCC diagnosis was collected.

### Statistical analysis

Categorical values were summarized using frequency and percentage and compared using the χ^2^ test. Continuous variables were summarized using median and interquartile range and compared using the Wilcoxon rank sum test. Piecewise logistic regression was used to investigate the yearly trends of HCC based on a breakpoint analysis. Coinciding with DAA introduction, 2014 was chosen to be the break point year for the regression analysis. Years were then divided into 2 eras: the pre-DAA era from 2009 to 2013 and the DAA era from 2014 to 2019. Odds ratios (ORs) and 95% confidence intervals (CIs) are reported for the change in yearly odds of an HCC patient having a given etiology. A *P* value of less than 0.05 was defined as statistically significant. All analyses were performed using SAS software, version 9.4 (SAS Institute; Cary, NC).

## RESULTS

### Study population

A total of 2,623 HCC cases were identified from the year 2009 to 2019 (Table 1), with 1,127 cases diagnosed in the pre-DAA era (2009–2013) and 1,496 cases diagnosed in the DAA era (2014–2019). The mean age of patients at the time of HCC diagnosis was 62 years (interquartile range [IQR] = 57–69). Most of the cases were male individuals (78%) and White (77%). At the time of HCC diagnosis, 87% of the cases was cirrhotic, 37% was obese, and 37% had diabetes. In the entire cohort, HCV alone or in combination with alcohol was the most common underlying etiology of HCC (53%), followed by NAFLD (17%) and alcoholic liver disease (13%) (Table 1).

**Table 1.** Characteristics of the patients with hepatocellular carcinoma

Variable	Overall	Period		*P* value
		2009–2013 N = 1,127	2014–2019 N = 1,496	
Age (yr)	62.0 (57.0, 69.0)	61.0 (56.0, 68.0)	63.0 (58.0, 69.0)	<0.0001
Male sex	2,046 (78.0%)	881 (78.2%)	1,165 (77.9%)	0.86
Race				
White	2,009 (76.9%)	881 (78.7%)	1,128 (75.6%)	0.18
Black	427 (16.3%)	177 (15.8%)	250 (16.7%)	
Asian	64 (2.4%)	23 (2.1%)	41 (2.7%)	
Other	32 (1.2%)	13 (1.2%)	19 (1.3%)	
Unknown	81 (3.1%)	26 (2.3%)	55 (3.7%)	
Hispanic ethnicity	81 (3.1%)	26 (2.3%)	55 (3.7%)	0.08
Center^a^				
Atrium Health	1,326 (50.6%)	523 (46.4%)	803 (53.7%)	0.0002
IU	1,297 (49.4%)	604 (53.6%)	693 (46.3%)	
BMI at HCC diagnosis	27.9 (24.3, 32.1)	27.8 (24.5, 31.9)	27.9 (24.3, 32.3)	0.94
Obesity	946 (36.5%)	396 (35.8%)	550 (37.0%)	0.56
Diabetes	969 (37.1%)	408 (36.3%)	561 (37.7%)	0.49
Hypertension	1,602 (61.3%)	680 (60.6%)	922 (61.8%)	0.50
Dyslipidemia	722 (27.6%)	259 (23.1%)	463 (31.1%)	<0.0001
Coronary artery disease	475 (18.2%)	211 (18.8%)	264 (17.7%)	0.48
Peripheral vascular disease	290 (11.1%)	95 (8.5%)	195 (13.1%)	0.0002
History of alcohol abuse	1,091 (42.4%)	508 (45.5%)	583 (40.0%)	0.0051
HIV-positive	33 (1.3%)	15 (1.3%)	18 (1.2%)	0.77
Total bilirubin (mg/dL)	1.2 (0.8, 2.1)	1.4 (0.9, 2.2)	1.1 (0.7, 2.0)	<0.0001
Albumin (g/dL)	3.3 (2.8, 3.7)	3.1 (2.7, 3.5)	3.4 (2.9, 3.8)	<0.0001
Platelets (K/cumm)	118.0 (78.0, 183.0)	111.0 (73.0, 169.5)	123.0 (84.0, 192.0)	<0.0001
Creatinine (mg/dL)	0.9 (0.7, 1.1)	0.9 (0.7, 1.2)	0.9 (0.7, 1.1)	0.55
INR	1.2 (1.1, 1.3)	1.2 (1.1, 1.4)	1.2 (1.1, 1.3)	0.0025
MELD score	10.0 (8.0, 14.0)	11.0 (9.0, 14.0)	10.0 (8.0, 14.0)	<0.0001
Complications				
Ascites	1,122 (42.8%)	583 (51.7%)	539 (36.0%)	<0.0001
Encephalopathy	737 (28.1%)	380 (33.7%)	357 (23.9%)	<0.0001
Varices	1,069 (40.8%)	503 (44.6%)	566 (37.8%)	0.0005
SBP	96 (3.7%)	56 (5.0%)	40 (2.7%)	0.0019
Portal vein thrombosis	529 (20.2%)	255 (22.6%)	274 (18.3%)	0.0065
Other	353 (13.5%)	236 (20.9%)	117 (7.8%)	<0.0001
No complications occurred	593 (22.6%)	168 (14.9%)	425 (28.4%)	<0.0001
Information not available or N/A	224 (8.5%)	82 (7.3%)	142 (9.5%)	0.0444
Cirrhotic status				
Cirrhotic	2,288 (87.2%)	1,001 (91.1%)	1,287 (87.0%)	<0.0001
Noncirrhotic	290 (11.1%)	98 (8.9%)	192 (13.0%)	
Unclassified	40 (1.5%)	28 (2.5%)	12 (0.8%)	
Missing entirely	5 (0.2%)	0 (0.0%)	5 (0.3%)	
Underlying etiology				
AIH/PBC/PSC	35 (1.3%)	11 (1.0%)	24 (1.6%)	0.07
Alcohol alone	331 (12.6%)	158 (14.0%)	173 (11.6%)	
HBV + alcohol	21 (0.8%)	7 (0.6%)	14 (0.9%)	
HBV alone	87 (3.3%)	38 (3.4%)	49 (3.3%)	
HC/A1ATD	30 (1.1%)	18 (1.6%)	12 (0.8%)	
HCV + alcohol	686 (26.2%)	313 (27.8%)	373 (24.9%)	
HCV + HBV (+/− alcohol)	37 (1.4%)	17 (1.5%)	20 (1.3%)	
HCV alone	698 (26.6%)	287 (25.5%)	411 (27.5%)	
NAFLD	451 (17.2%)	178 (15.8%)	273 (18.2%)	
Rare etiologies	14 (0.5%)	8 (0.7%)	6 (0.4%)	
Unclear/unknown	233 (8.9%)	92 (8.2%)	141 (9.4%)	

A1ATD, alpha-1-antitrypsin deficiency; AIH, autoimmune hepatitis; HBV, hepatitis B virus; BMI, body mass index; HCV, hepatitis C virus; HC, hemochromatosis; INR, international normalized ratio; IU, Indiana University; MELD, Model for End-Stage Liver Disease; N/A, not applicable; NAFLD, nonalcoholic fatty liver disease; PBC, primary biliary cholangitis; PSC, primary sclerosing cholangitis; SBP, spontaneous bacterial peritonitis.

a Categorical values were summarized using frequency and percentage, and continuous variables were summarized using median and interquartile range.

### HCC trends by underlying liver etiology

**Figure 1.**
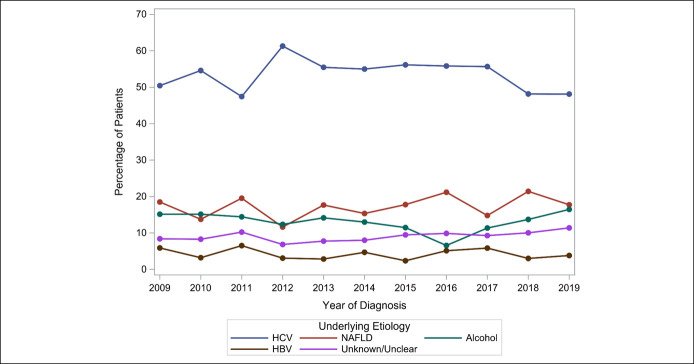
Ten-year trend of underlying etiology associated with hepatocellular carcinoma. HBV, hepatitis B virus; HCV, hepatitis C virus; NAFLD, nonalcoholic fatty liver disease.

**Figure 2.**
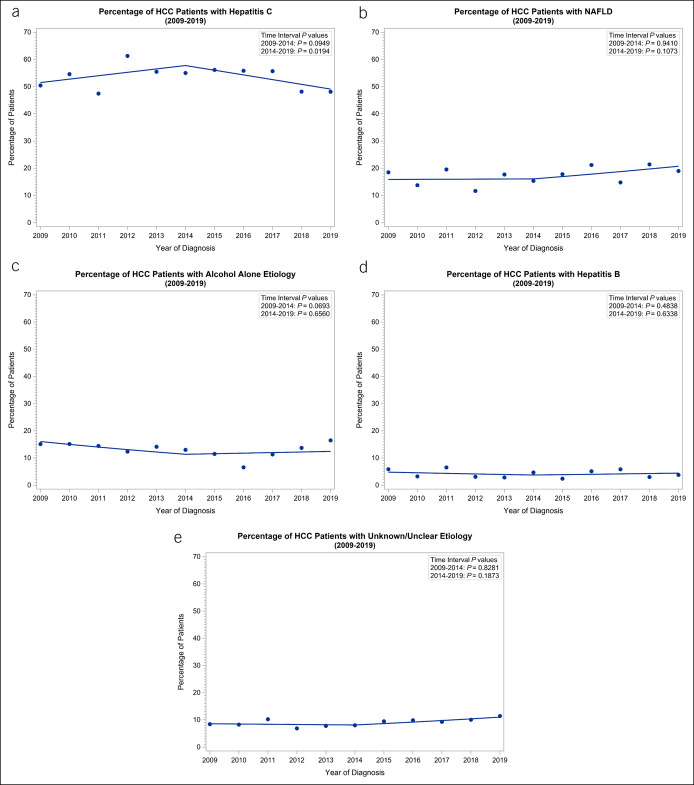
Patient breakdown of liver-associated diseases with hepatocellular carcinoma, 2009–2019. (a) hepatitis C (b) nonalcoholic fatty liver disease (c) alcohol (d) hepatitis B (e) unknown.

Over the course of the 10-year study period, HCV remained the dominant liver disease associated with HCC, followed by NAFLD and alcohol (Figure 1). Using 2014 as a break point for the DAA era, a significant annual decline in HCV-HCC cases in the DAA era was observed (OR = 0.93, 95% CI 0.88–0.99, *P* = 0.019) (Figure 2a). There were no significant changes in the trends of NAFLD-related, alcohol-related, or HBV-related HCC in the 2 eras (Figure 2b–e).

### Trends in HCV treatment and response

Compared with the pre-DAA era, the percentage of patients with HCV who did not receive HCV therapy significantly decreased in the DAA era (59% vs 35%, *P* < 0.001) (Table 2). DAA therapy was given to a total of 516 (36%) of 1,421 patients with HCV from both eras but at much higher rate in the post-DAA era (51% vs 17%, *P <* 0.0001). The rates of SVR achieved were significantly higher in the patients diagnosed with HCC and treated for HCV in the DAA era (79% vs 64%, *P <* 0.0001). Of note, 19% of cases with HCV still received interferon in the post-DAA era, probably reflecting early challenges in implementing DAA therapy in practice. There were no differences in types of HCV treatment received (DAA or interferon-based regimen) between noncirrhotic and cirrhotic HCV-HCC groups in the overall cohort (see Supplementary Data Table 1, http://links.lww.com/CTG/A715).

**Table 2.** HCV treatment regimens offered to patients with HCC and SVR, stratified according to DAA era

	Period		*P* value
	2009–2013 N = 617	2014–2019 N = 804	
HCV treatment received before or after HCC diagnosis?			
After	81 (13.1%)	137 (17.1%)	<0.0001
Before	145 (23.5%)	362 (45.0%)	
Not applicable^a^	391 (63.4%)	305 (37.9%)	
Treatment			
DAA	107 (17.3%)	409 (50.9%)	<0.0001
Pegylated interferon	153 (24.8%)	156 (19.4%)	0.0151
Ribavirin	159 (25.8%)	202 (25.2%)	0.79
Unknown	32 (5.2%)	64 (8.0%)	0.0384
No treatment received	366 (59.3%)	282 (35.1%)	<.0001
SVR	143 (64.1%)	391 (78.8%)	<.0001

DAA, Direct-acting antiviral; HCV, hepatitis C virus; HCC, hepatocellular carcinoma; SVR, sustained virological response.

a Patients who did not receive treatment, unknown treatment type, or patients who may have multiple treatments both before and after HCC diagnosis.

Note: The exact date of HCV treatment receipt relative to HCC diagnosis is not known because it could not be collected from the records of many patients.

### HCC trends by cirrhotic status

**Figure 3.**
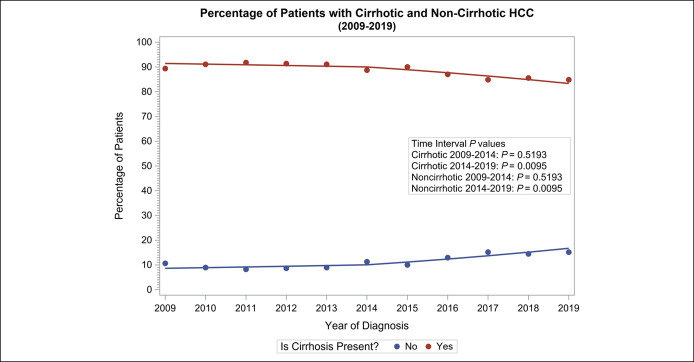
Percentage of patients with cirrhotic and noncirrhotic hepatocellular carcinoma, 2009–2019.

Of the 290 noncirrhotic HCC cases in the entire cohort, most of the cases (66%) were diagnosed in the DAA era. There was no change in the annual trends of noncirrhotic HCC cases in the pre-DAA era but an annual increase in noncirrhotic HCC cases was observed in the DAA era (OR 1.13, 95% CI 1.03–1.23, *P* = 0.0095) (Figure 3). There was also a significant decline in cirrhotic HCC in the DAA era (OR 0.89, 95% CI 0.81–0.97, *P =* 0.0095).

### Trends of cirrhotic status of HCC by underlying liver disease

**Figure 4.**
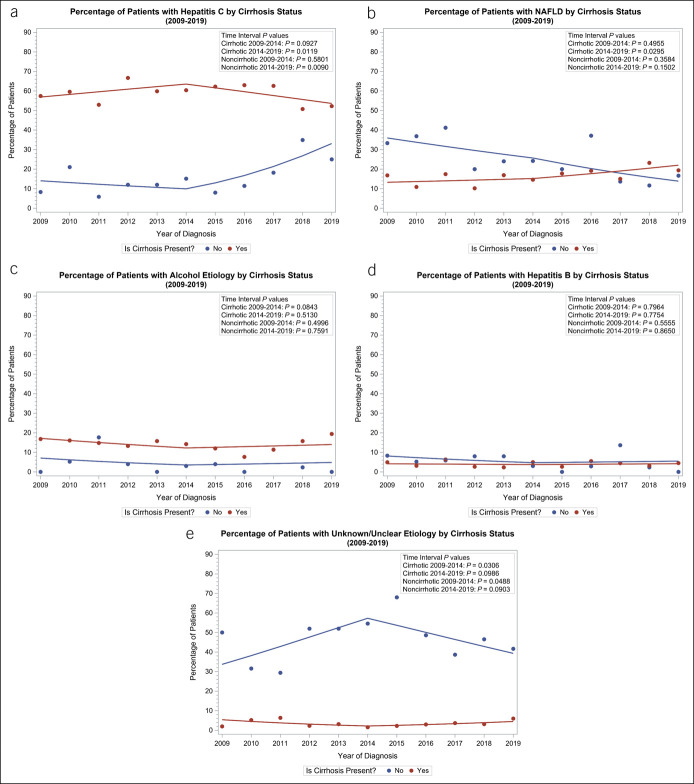
Cirrhosis status and liver-associated disease breakdown. (**a**) hepatitis C (**b**) nonalcoholic fatty liver disease, (**c**) alcohol, (**d**) hepatitis B, (**e**) unknown.

For HCV-HCC, 2 significant changes in trends of cirrhotic status were observed in the DAA era: annual decrease in cirrhotic cases (OR 0.92, 95% CI 0.86–0.98, *P* = 0.012) and annual increase in noncirrhotic cases (OR 1.35, 95% CI 1.08–1.69, *P =* 0.009) (Figure 4a). For NAFLD, an annual increase in cirrhotic cases in the DAA era was observed (OR 1.10, 95% CI 1.01–1.19, *P* = 0.03), whereas noncirrhotic NAFLD-HCC did not show significant change in trend (Figure 4b). There were no significant changes in trends of alcohol-related or HBV-related cirrhotic and noncirrhotic HCC cases in either of the 2 eras (Figure 4c–e).

### Differences in HCC tumor characteristics between DAA and pre-DAA eras

The mean tumor size at the time of HCC diagnosis was slightly larger in the DAA era compared with that in the pre-DAA era (3.7 cm vs 3.5 cm, *P* = 0.039) (Table 3). There were significant differences in the percentages of tumor differentiations levels between the 2 eras (*P =* 0.0002). In the DAA era, well-differentiated HCC was less frequent (25% vs 36%), whereas moderately differentiated HCC (54% vs 48%) and poorly differentiated HCC (20% vs 15%) were more frequent. There were no significant differences between the 2 eras in anatomic staging of lesions, number of HCC lesions, or tumors within the Milan criteria at the time of HCC diagnosis (Table 3). However, higher percentages of patients presented in Barcelona Clinic Liver Cancer stages A and B in the DAA era (50% vs 30%) vs the pre-DAA era (*P* < 0.0001).

**Table 3.** Tumor characteristics in the DAA and Pre-DAA era

Variable	Period		*P* value
	2009–2013 N = 1,127	2014–2019 N = 1,496	
Tumor size (cm)	3.5 (2.2, 5.8)	3.7 (2.4, 6.5)	0.0385
AFP category			
<20	506 (48.2%)	738 (52.7%)	0.05
20–200	224 (21.3%)	292 (20.9%)	
>200	320 (30.5%)	370 (26.4%)	
Tumor differentiation			
Well	189 (36.0%)	171 (24.8%)	0.0002
Moderate	251 (47.8%)	370 (53.7%)	
Poor	77 (14.7%)	139 (20.2%)	
Undifferentiated/anaplastic	8 (1.5%)	9 (1.3%)	
Anatomic stage category			
Stage I or II	512 (66.3%)	863 (66.0%)	0.87
Stage III or IV	260 (33.7%)	445 (34.0%)	
Tumor stage			
Single	483 (43.0%)	639 (43.0%)	0.68
3 tumors < 3 cm	139 (12.4%)	168 (11.3%)	
Large multinodular	225 (20.0%)	322 (21.7%)	
Vascular invasion or extrahepatic spread	277 (24.6%)	358 (24.1%)	
Tumor within Milan criteria	540 (48.0%)	677 (45.5%)	0.20
BCLC stage			
Stage A	171 (20.9%)	528 (36.4%)	<0.0001
Stage B	76 (9.3%)	193 (13.3%)	
Stage C	382 (46.6%)	530 (36.5%)	
Stage D	190 (23.2%)	200 (13.8%)	

AFP, α-fetoprotein; BCLC, Barcelona Clinic Liver Cancer; DAA, direct-acting antiviral.

### Trends of HCC treatment modalities utilization

In the DAA era, more HCC cases underwent resection (14% vs 11%, *P =* 0.013) and less underwent transplantation (12% vs 15%, *P =* 0.023) (Table 4), corresponding to the increased number of noncirrhotic HCC cases during the same period. For HCV-HCC, rate of resection increased (5% vs 11%, *P* = 0.0002) and rate of transplantation decreased (19% vs 12%, *P* = 0.0006) in the DAA era compared with the pre-DAA era. When comparing cirrhotic and noncirrhotic HCV-HCC cases, higher rates of resection and zero cases of transplantation were noted in noncirrhotic HCC group (Table S2). There were no differences in trends of utilization of liver-directed or ablative therapies between the 2 eras.

**Table 4.** Treatment modalities offered to patients with HCC, stratified according to DAA era

Variable	Period		*P* value
	2009–2013 N = 1,127	2014–2019 N = 1,496	
Treatment modalities			
Resection	121 (10.7%)	209 (14.0%)	0.0134
Resection for those with HCV-related HCC	33 (5.4%)	87 (10.8%)	0.0002
Liver transplantation (all)	170 (15.1%)	180 (12.0%)	0.0229
Liver transplantation for those within Milan criteria	139 (25.7%)	146 (21.6%)	0.09
Liver transplantation for those with HCV-related HCC	114 (18.5%)	96 (11.9%)	0.0006
Catheter-delivered therapy	555 (49.2%)	701 (46.9%)	0.23
Sorafenib	202 (17.9%)	190 (12.7%)	0.0002
SBRT	100 (8.9%)	130 (8.7%)	0.87
RFA and/or microwave ablation	183 (16.2%)	271 (18.1%)	0.21
Palliative/Hospice care	402 (35.7%)	498 (33.3%)	0.20
Other	40 (3.5%)	103 (6.9%)	0.0002
None	57 (5.1%)	44 (2.9%)	0.0053
Unknown	19 (1.7%)	26 (1.7%)	0.92

DAA, direct-acting antiviral; HCV, hepatitis C; HCC, hepatocellular carcinoma; RFA, radiofrequency ablation; SBRT, stereotactic body radiation therapy.

Decreased utilization of sorafenib was observed in the DAA era (13% vs 18%, *P =* 0.0002) coinciding with an increase in utilization of newer therapies for HCC such as nivolumab (categorized as other) (7% vs 4%, *P* = 0.0002).

### Trends of cirrhosis-related complications

The rates of hepatic complications including decompensation events and portal venous thrombosis in all cirrhotic HCC cases were significantly lower in the DAA era than in the pre-DAA era (Table 1). The same findings were observed when only cirrhotic HCV-HCC cases were assessed based on receipt of DAA (Table S3).

## DISCUSSION

Although HCV remains the dominant liver disease associated with HCC, this study showed significant decline in the proportions of HCV-HCC cases since the introduction of DAA. Of importance, the overall noncirrhotic HCC cases increased whereas cirrhotic HCC cases decreased in the DAA era, mainly driven by similar trends in noncirrhotic and cirrhotic HCV-HCC in the same period. These changes were associated with increased utilization of resection and decreased utilization of liver transplantation for HCC treatment, also driven by similar changes in utilization of these treatment modalities for HCV-HCC in the DAA era.

The decline in HCC incidence in patients with HCV achieving SVR with DAA therapy is beginning to have an impact on the burden of HCV-HCC (12,15,18). This trend is associated with a change in the mix of cirrhotic and noncirrhotic HCV-HCC cases. The annual increase in noncirrhotic HCC cases in the DAA era in this study was largely driven by an increase in noncirrhotic HCV-HCC cases. There were no differences in the type or frequency of treatment for HCV (DAA or interferon-based) between noncirrhotic and cirrhotic HCV-HCC cases to suggest undertreatment of noncirrhotic cases causing their uptrend in the DAA era.

As expected, more cases with HCC received HCV therapy and achieved SVR in the DAA era. However, between 2014 and 2019, only 50% of cases with HCV were treated with DAAs, and the SVR rates achieved in this population (79%) were lower than SVR rates that exceed 90% in the non-HCC setting (7,8). This is consistent with the findings of a recent meta-analysis that showed a significant reduction in pooled SVR rate in patients with HCC compared with non-HCC patients and a large drop in SVR rate to 73% in cases with active HCC (32). These findings along with the continued use of interferon in the early DAA era reflect a transition phase to the newer therapeutic agents and highlight the early challenges of rapidly changing composition and duration of DAA combination regimens, practice patterns, and HCV patient's selection for DAA therapy in that period (19,33,34).

Liver transplantation utilization decreased whereas resection for HCC increased in the DAA era in this study, driven by similar utilization trended for these modalities for HCV-HCC in the same period. Other studies have yielded similar results indicating the positive impact of DAA in reducing model of end-stage liver disease score and decreased listing for and utilization of liver transplantation in HCV-HCC (35–37). Puighevi et al. recently described a 14.6% decrease in liver transplantation utilization for HCV-HCC in the DAA era (38). Recent studies have also noted an alarming trend of increased liver transplantation rates on NAFLD-HCC cases (39,40). But to our knowledge, no other study reported increasing resection rates for HCV-HCC in the DAA era.

We previously reported a downtrend in noncirrhotic HCC and an uptrend for cirrhotic HCC between 2000 and 2014 (22). As we moved into the DAA era in this study, these trends seem to flip with rising noncirrhotic HCC and declining cirrhotic HCC between 2014 and 2019. The new trends are mainly due to changes in cirrhotic and noncirrhotic HCV-HCC in the DAA era. Of importance, the earlier significant uptrend in cirrhotic NAFLD-HCC we observed between 2000 and 2014 has continued through 2019, highlighting the enlarging contribution of NAFLD to HCC burden. This is a particularly important trend and supports future projections for NAFLD overtaking HCV as the primary etiology of HCC in the DAA era (41,42).

This study has a few limitations. It was conducted at tertiary care centers, and its findings may not generalize to other practice settings. Owing to its retrospective nature, some data points were missing. We could not determine the trends of HCC related to autoimmune and rare etiologies because of the small numbers in our data set. Another limitation to our study is that it involved only 2 tertiary care centers where liver resection and transplant are available options for HCC treatment. This contrasts with nontransplant centers where resection, rather than transplant, may be used more as the locally available option to some patients with HCC. Furthermore, changing resection and transplant practices for HCC across different regions in the United States may limit the generalization of pattern of utilization for these modalities we observed in our study to the rest of the United States. The study also has several strengths. The 10-year study period helped us examine the trends of HCC at our 2 sites. With 5-year data available pre-2014 and post-2014, we were able to evaluate the impact of DAA therapy in its early years of introduction on trends of liver disease etiologies and cirrhotic status in cases with HCC. We collected detailed patient-level data that allowed ascertainment of underlying liver disease and cirrhotic status and assessment of changes in utilization of different HCC treatment modalities before and after DAAs introduction.

In conclusion, this study showed a significant decrease in HCV-HCC cases in the DAA era without change in the contribution of other liver disease to the overall burden of HCC. The new trends of increase in noncirrhotic-HCC and decrease in cirrhotic HCC in the DAA era were associated with increased utilization of resection and decreased utilization of liver transplantation for HCC treatment. How expanded access to and changing patterns of DAAs use in practice will further affect the contribution of HCV to the overall burden of HCC, proportions of noncirrhotic HCC, and utilization of HCC different treatment modalities will require further study.

## CONFLICTS OF INTEREST

**Guarantor of article**: Samer Gawrieh, MD.

**Specific author contributions**: K.M.: data collection, data analysis, manuscript preparation, and manuscript revision. A.M., M.P., L.D.: data collection and manuscript revision. H.B., H.L.: data analysis and manuscript revision. L.N., N.C., A.D.: study design, data analysis and manuscript revision. S.G.: study design, data analysis, manuscript preparation and manuscript revision.

**Financial support:** Internal funds from the Division of Gastroenterology and Hepatology at Indiana University School of Medicine.

**Potential competing interests:** K. Mathur, A. Mazhar, M. Patel, L. Dakhoul, H. Heather Burney, H. Liu and L. Nephew have no conflicts of interest or financial disclosures. N. Chalasani has ongoing research support from Eli Lilly, Galectin Therapeutics, Intercept, and Exact Sciences, In the past 12 months, he has received consulting fees from Abbvie, Madrigal, Nusirt, Allergan, Siemens, Genentech, Zydus, La Jolla, Axcella, Foresite Labs, and Galectin Therapeutics. A. deLemos has served on an advisory board for Takeda Pharmaceuticals in the last 12 months. S. Gawrieh consulting: TransMedics, Pfizer. Research grant support: Cirius, Galmed, Viking, and Zydus.

**Study Highlights**

**WHAT IS KNOWN**

✓ Direct-acting antivirals (DAA) decrease hepatocellular carcinoma (HCC) risk in patients with hepatitis C virus (HCV).
✓ HCV and nonalcoholic fatty liver disease are leading causes of HCC in the United States.

**WHAT IS NEW HERE**

✓ A trend of increase in noncirrhotic HCC and decrease in cirrhotic HCC was observed in the DAA era (following the year 2014).
✓ A driving factor is similar increase in noncirrhotic HCC and decrease in cirrhotic HCV-HCC in the DAA era.
✓ The rise in cirrhotic nonalcoholic fatty liver disease-HCC observed in the pre-DAA era continued in the DAA era.
✓ Rates of resection increased whereas utilization of transplantation for HCC decreased in the DAA era.

## Supplementary Material

SUPPLEMENTARY MATERIAL
