# Supplementary material for: Changing Trends of Cirrhotic and Noncirrhotic Hepatocellular Carcinoma in the Era of Directly-Acting Antiviral Agents
Source: Clin Transl Gastroenterol. 2021 Nov 3;12(11):e00420. doi: 10.14309/ctg.0000000000000420 (PMC8568358; doi:10.14309/ctg.0000000000000420)
Supplement: SUPPLEMENTARY MATERIAL [file ct9-12-e00420-s001.docx]

**Supplementary Tables**

**Table S1: HCV treatment and SVR rates in cirrhotic and non-cirrhotic HCV-related HCC:**

| **HCV Treatment Type** | **Overall N=1,413** | **Non-Cirrhotic N=49** | **Cirrhotic N=1,364** | **p-value** |
| --- | --- | --- | --- | --- |
| **DAA** | 516 (36.5%) | 20 (40.8%) | 496 (36.4%) | 0.53 |
| SVR Attributed to DAA | 437 (84.7%) | 17 (85.0%) | 420 (84.7%) | 0.97 |
| **Interferon** | 309 (21.9%) | 16 (32.7%) | 293 (21.5%) | 0.06 |
| SVR Attributed to IFN | 62 (20.1%) | 6 (37.5%) | 56 (19.1%) | 0.07 |

**Table S2: Treatment modalities for cirrhotic and non-cirrhotic HCV related HCC:**

| **Treatment Modality** | **Non-Cirrhotic HCV N=49** | **Cirrhotic HCV N=1,364** | **p-value** |
| --- | --- | --- | --- |
| Resection | 22 (44.9%) | 97 (7.1%) | <.0001 |
| Liver Transplantation | 0 (0.0%) | 210 (15.4%) | 0.0029 |
| Transplant within Milan Criteria | 0 (0.0%) | 164 (23.0%) | 0.0148 |
| Catheter Delivered Therapy | 22 (44.9%) | 682 (50.0%) | 0.48 |
| Sorafenib | 9 (18.4%) | 189 (13.9%) | 0.37 |
| Radiation (SBRT) | 3 (6.1%) | 131 (9.6%) | 0.41 |
| RFA and/or Microwave Ablation | 8 (16.3%) | 279 (20.5%) | 0.48 |
| Palliative/Hospice Care | 2 (4.1%) | 473 (34.7%) | <.0001 |
| Other | 5 (10.2%) | 63 (4.6%) | 0.07 |
| None | 0 (0.0%) | 58 (4.3%) | 0.14 |
| Unknown | 0 (0.0%) | 22 (1.6%) | 0.37 |

**Table S3: Complication rates in cirrhotic HCV- related HCC cases per DAA treatment receipt:**

| **Complication** | **Treated with DAA** | | **p-value** |
| --- | --- | --- | --- |
|  | **No N=904** | **Yes N=516** |  |
| Ascites | 463 (51.2%) | 185 (35.9%) | <.0001 |
| Encephalopathy | 298 (33.0%) | 145 (28.1%) | 0.06 |
| Varices | 390 (43.1%) | 233 (45.2%) | 0.46 |
| SBP | 40 (4.4%) | 11 (2.1%) | 0.0255 |
| Other | 165 (18.3%) | 34 (6.6%) | <.0001 |
| No complications occurred | 170 (18.8%) | 173 (33.5%) | <.0001 |
| Information not available or N/A | 32 (3.5%) | 17 (3.3%) | 0.81 |
| Portal vein thrombosis | 232 (25.7%) | 66 (12.8%) | <.0001 |
